# Severity of COVID-19 infection in patients with COVID-19 combined with diabetes

**DOI:** 10.1186/s41043-024-00548-w

**Published:** 2024-04-23

**Authors:** Dan Lu, Yuhong Liu, Pengcheng Ma, Rui Hou, Jin Wang

**Affiliations:** Department of Radiology, Yan’an Hospital Affiliated to Kunming Medical University, No. 245 Renmin East Road, Panlong District, 650051 Kunming, Yunnan China

**Keywords:** COVID-19, Diabetes, Poor blood glucose control, Tomography, Computed tomography severity score

## Abstract

**Purpose:**

This study aimed to analyse the correlation between blood glucose control and the severity of COVID-19 infection in patients with diabetes.

**Methods:**

Clinical and imaging data of a total of 146 patients with diabetes combined with COVID-19 who visited our hospital between December 2022 and January 2023 were retrospectively collected. The patients were divided into the ‘good blood glucose control’ group and the ‘poor blood glucose control’ group based on an assessment of their blood glucose control. The clinical data, computed tomography (CT) appearance and score and the severity of COVID-19 infection of the two groups were compared, with the severity of COVID-19 infection being the dependent variable to analyse other influencing factors.

**Results:**

The group with poor blood glucose control showed a higher lobar involvement degree and total CT severity score (CTSS) than the group with good blood glucose control (13.30 ± 5.25 vs. 10.38 ± 4.84, *p* < 0.05). The two groups exhibited no statistically significant differences in blood lymphocyte, leukocyte, C-reaction protein, pleural effusion, consolidation, ground glass opacity or crazy-paving signs. Logistic regression analysis showed that the total CTSS significantly influences the clinical severity of patients (odds ratio 1.585, *p* < 0.05), whereas fasting plasma glucose and blood glucose control are not independent factors influencing clinical severity (both *p* > 0.05). The area under the curve (AUC) of CTSS prediction of critical COVID-19 was 0.895 with sensitivity of 79.3% and specificity of 88.1% when the threshold value is 12.

**Conclusion:**

Blood glucose control is significantly correlated with the CTSS; the higher the blood glucose is, the more severe the lung manifestation. The CTSS can also be used to evaluate and predict the clinical severity of COVID-19.

## Introduction

The 2019 novel coronavirus pneumonia (COVID-19) is an infectious disease caused by the SARS-CoV-2 virus, which is highly susceptible to mutation [1]. Most patients contracting the virus experience mild to moderate respiratory diseases and can recover with symptomatic treatment. A relatively small number develop acute respiratory distress syndrome, multiple organ failure or even death, as observed mainly in older patients with underlying medical conditions [2]. The evidence suggests that elderly patients with chronic diseases such as hypertension, carotid artery diseases and diabetes are more susceptible to COVID-19 [3–5], and according to China’s census data, nearly half of the older population (in which the average life expectancy is continually increasing) suffer from diabetes [6].

Previous research has shown that, compared with normal infected individuals, patients with diabetes exhibit more severe conditions in terms of clinical course and chest imaging and are more likely to develop critical COVID-19 [5, 7, 8]. In addition, those with poor blood glucose control have a worse prognosis [9]. Diabetes has become a major risk factor for poor prognosis in patients with COVID-19 [10]. The severity of diabetes is associated with a rise in inflammation biomarker levels, leukocytosis and neutrocytophilia and is also an independent factor associated with the death of patients [11]. Therefore, it is necessary to carry out an in-depth exploration of the correlation between blood glucose control and the severity of lung infection to guide the clinical application of medication and promote prevention publicity, thereby improving patients’ quality of life.

## Materials and methods

### Research participants

A total of 146 patients with diabetes combined with COVID-19, including 87 men and 59 women, who visited the researchers’ hospital between December 2022 and January 2023 were retrospectively identified. According to their fasting plasma glucose (FPG) and glycated haemoglobin (HbA1c) levels upon admission, the patients were divided into the ‘good blood glucose control group’ (PFG ≤ 7 mmol/L and HbA1c < 7.0%) and the ‘poor blood glucose control group’ (PFG > 7 mmol/L or HbA1c ≥ 7.0%), which consisted of 53 and 93 patients, respectively. Based on the latest version of the Guidelines on Diagnosis and Treatment of Novel Coronavirus Pneumonia [4], the two groups of patients were clinically classified into common and critical types.

The inclusion criteria were as follows: (1) The reverse transcription polymerase chain reaction (RT-PCR) tested positive for COVID-19; (2) the latest recognised diabetes diagnostic criteria [7] were met: the FPG level measured in venous blood was ≥ 7 mmol/L, 2 h postload or random blood glucose ≥ 11.1 mmol/L and HbA1c ≥ 6.5%.

The exclusion criteria were as follows: (1) Previous history of lung tumours or lung surgery; (2) previous history of pulmonary tuberculosis, chronic obstructive pulmonary disease, pulmonary interstitial fibrosis or heart failure; (3) re-positive result in the RT-PCR test; (3) poor image quality that was inappropriate for evaluation.

### Clinical data

Patients’ basic information, including gender, age, white blood cell and lymphocyte counts, C-reactive protein (CRP) level, PFG, HbA1c and chest computed tomography (CT) scan images within 2 days of admission, was collected.

### Imaging data

#### Computed tomography scan

A plain chest CT scan from the apex to the base of the lungs was performed during the end-inhale phase by Philips 64-slice spiral CT. The parameters of the scan were as follows: tube voltage– 120 kV; tube current– 150 mA; slice thickness– 5 mm; spiral pitch– 1.0; lung window– (width 1,400 Hu, level − 600 Hu); mediastinal window– (width 400 Hu; level 40 Hu).

The CT severity score (CTSS) was employed to evaluate the severity of patients’ images and was based on the degree of lobar involvement (0–25). Image processing: The CT images of all cases were evaluated by two radiologists at a level of attending physician or above (without knowing clinical information) to determine whether there was (1) ground glass opacity (GGO); (2) consolidation; (3) pleural effusion; (4) crazy-paving signs. All CT images were uploaded to the Deepwise MetAI system to quantitatively score the affected area of each lung lobe against the following criteria: 0 affected (score 0), < 5% affected (score 1); 5–25% affected (score 2); 26–49% affected (score 3); 50–75% affected (score 4) and > 75% affected (score 5). Each lung lobe scores 0–5, giving a total score of 0–25.

### Statistical analysis

Statistical analysis was performed using the SPSS 26.0 software, where categorical variables were expressed as frequencies and percentages, continuous variables were expressed as mean values and quantitative data with non-normal distribution were calculated by quartiles. The chi-squared test was used to analyse categorical variables, the *t*-test for continuous variables with normal distribution and the rank sum test for those with non-normal distribution. Logistic regression analysis was performed to detect independent factors influencing clinical severity. A *p*-value of < 0.05 was considered statistically significant.

## Results

### Clinical data comparison

A total of 146 cases were included in this study, with an average age of 74 (65–83) years. Table 1 shows the statistical results of the clinical data of the two groups of patients. Specifically, the good blood glucose control group and poor blood glucose control group show no statistically significant differences (*p* ≥ 0.05) in gender, age, blood lymphocyte, leukocyte and CRP; the two groups showed a statistically significant difference in clinical classification, with the number of critically ill patients in the poor blood glucose control group higher than those in the good blood glucose control group. The group with poor blood glucose control showed a higher lobar involvement degree and total CTSS than the group with good blood glucose control (13.30 ± 5.25 vs. 10.38 ± 4.84, *p* < 0.05).

**Table 1:** Clinical data of two groups

Variable	Good blood glucose control group (*N* = 53)	Poor blood glucose control group (*N* = 93)	Statistics	*P*
Age/Year	74(66.5 ∼ 83.5)	74(64.5 ∼ 83)	-0.499(z)	0.618
Sex/Case			1.578(c2)	0.209
Male	28(53%)	59(63%)		
Female	25(47%)	34(37%)		
CRP	18.91(4.53 ∼ 96.88)	44.12(15.71 ∼ 107.16)	-1.960(z)	0.050
Leukocyte	5.75(4.71 ∼ 7.16)	6.84(4.81 ∼ 8.5)	-2.055(z)	0.400
Lymphocyte	1.02(0.75 ∼ 1.32)	0.96(0.61 ∼ 1.31)	-0.669(z)	0.500
Clinical classification			5.329(c2)	0.021
Common	28(53%)	31(33%)		
Critical	25(47%)	62(67%)		

Note: No data in the table conforms to a normal distribution and all data were showed as median and IQR. Gender and clinical classification were analyzed by chi-square test; age, CRP, leukocyte and lymphocyte were tested by rank sum test

### Computed tomography appearance and score comparison

**Fig. 1:**
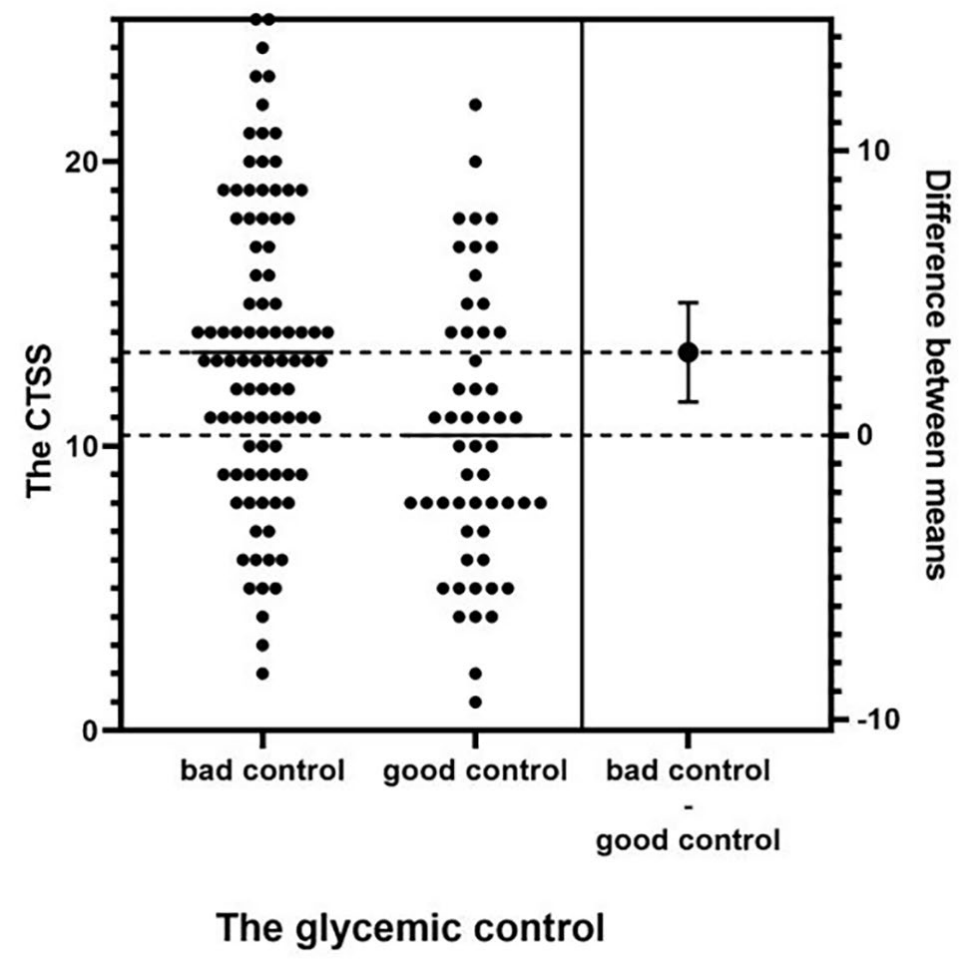
CTSS score in bad control and good control of blood group and the diffrerence between two group

The two groups of patients exhibited no statistically significant differences (*p* > 0.05) in the presence of pleural effusion, consolidation, GGO and crazy-paving signs in CT images, although the scores of lung lobes were significantly different across the two groups (Table 2). The CTSS and FPG level were significantly positively correlated, with *p* = 0.007, correlation coefficient *r* = 0.221 and a moderate level of correlation; the total CTSS and diabetes blood glucose control were significantly positively correlated, with *p* = 0.001and *r* = 0.268, indicating a significantly positive correlation and a moderate level of correlation (Fig. 1).

**Table 2:** Chest CT index of the two groups

Variable	Good blood glucose control group (*N* = 53)	Poor blood glucose control group (*N* = 93)	Statistics	*P*
CTSS score/score	10.38 ± 4.84	13.30 ± 5.25	-3.326①	0.001
Lung lobe score/score				
Left upper lobe	2(1 ∼ 2.5)	2(1 ∼ 3)	-2.790②	0.005
Left lower lobe	3(2 ∼ 4)	3(2 ∼ 4)	-2.363②	0.118
Right upper lobe	2(1 ∼ 2)	2(1 ∼ 3)	-2.661②	0.008
Right middle lobe	1(1 ∼ 2)	2(1 ∼ 3)	-2.736②	0.006
Right lower lobe	3(2 ∼ 4)	3(2 ∼ 4)	-2.567②	0.010
Pleural effusion/case			0.643③	0.423
Yes	17(32%)	36(39%)		
No	36(68%)	57(61%)		
Consolidation/case			1.467③	0.226
Yes	30(57%)	62(67%)		
No	23(43%)	31(33%)		
Ground glass opacity/case			2.567③	0.109
Yes	40(75%)	80(86%)		
No	13(25%)	13(14%)		
Crazy-paving sign/case			3.557③	0.590
Yes	15(28%)	41(44%)		
No	38(72%)	52(56%)		

Note: No data in the table conforms to a normal distribution and data were showed as median and quartiles ① Independent sample t test; ② Rank sum test; ③ Chi-square test

### Logistic regression analysis of independent factors influencing clinical severity

**Fig. 2:**
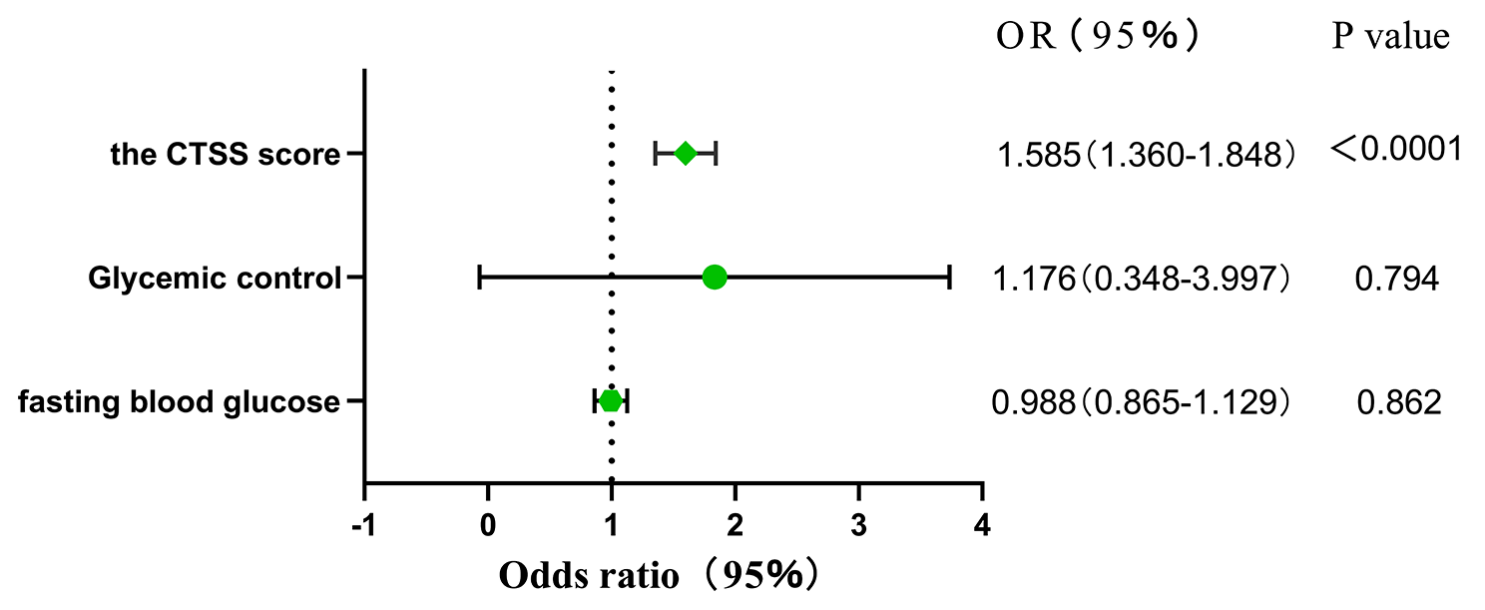
Logistic regression analysis of independent factors influencing clinical severity. The medel was adjusted for age, gender, HbA1c, FPG, CTSS and CRP

The total CTSS significantly influences patients’ clinical severity, *p* < 0.05. Further, the influence coefficient is 0.461, indicating that the higher the total CTSS is, the more likely the patients are to have a critical clinical manifestation. For every 1-point increase in the total CTSS, the patients are 1.585 times more likely to exhibit a critical clinical manifestation. Patients’ FPG level and blood glucose control are not independent factors influencing clinical severity, *p* > 0.05 (Fig. 2).

### Computed tomography severity score evaluation and prediction of COVID-19 clinical classification

**Fig. 3:**
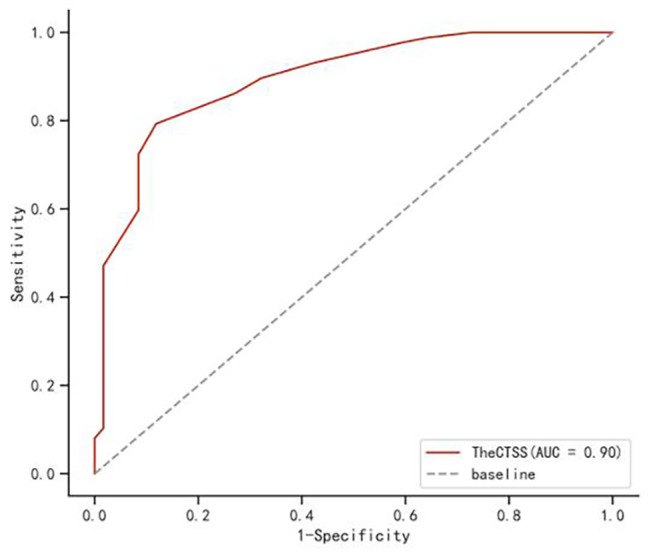
The ROC curve of clinical classification prediction by CTSS score

The area under the curve (AUC) of CTSS prediction of critical COVID-19 was 0.895 with sensitivity of 79.3% and specificity of 88.1% (Fig. 3) when the threshold value is 12 [12].

## Discussion

Our results showed that chest CT imaging is a reliable testing method for COVID-19 evaluation; the higher the blood glucose level is, the more evident the lung inflammation and the more severe the clinical manifestation.

The proportion of patients with diabetes has been growing in the older population [13]. High blood glucose causes an increase of dicarbonyl molecules in the human body, inhibits the antibacterial and immune function of β-defensins and increases the susceptibility of patients, particularly among those with uncontrolled or poorly controlled blood glucose [14]. Previous studies have shown that high blood glucose reduces peripheral blood lymphocytes and increases leukocytes in patients with COVID-19, leading to immune system overactivation and more severe clinical symptoms [5, 15]. Although some of the research indicates that neutrophils and leukocytes help distinguish asymptomatic and moderate COVID-19 [16], this study found that the differences in lymphocytes and leukocytes among patients are not statistically significant.

According to the latest version of the Guidelines on Diagnosis and Treatment of Novel Coronavirus Pneumonia, the diagnostic criterion for COVID-19 is a positive RT-PCR test; however, the detecion takes about one day and the production of test kits is limited. Comparatively, a chest CT scan is an important tool for the rapid screening and initial diagnosis of COVID-19; given its convenience, it can serve as a predictive indicator to evaluate illness severity and clinical prognosis [12, 17, 18] and help in the timely adjustment of patients’ diagnosis and treatment [19]. Most patients show abnormal manifestations of the lungs 2–3 days after contracting COVID-19. In the early stage of the disease, the virus tends to invade peripheral blood vessels and bronchioles, causing interstitial changes in lung tissue, such as interstitial inflammatory oedema and interlobular septal thickening, leading to a rise in pressure of lung parenchyma and exudation of fibrinous and high-protein mucus within bronchiole and thus the formation of GGO and halo sign in the subpleural lung region. Over time, interlobular septal thickening further causes the formation of crazy-paving signs, restricting the absorption of alveolar exudate and causing alveolar consolidation and in serious cases, even diffuse alveolar damage, eventually leading to a white lung and pleural effusion in some patients [18, 20, 21]. A review of relevant literature reveals that compared with patients without diabetes, patients with diabetes show more evident inflammation on chest CT and those with poor blood glucose control exhibit more severe lung damage [3, 5]. Similar to previous studies, the majority of patients in this experiment demonstrated GGO and lung consolidation on their CT appearance, and a few even showed pleural effusion and crazy-paving signs. However, the differences in these changes are statistically insignificant between the two groups, which may be attributed to the small number of cases and good treatment received by most patients during the early onset of the disease.

**Fig. 4:**
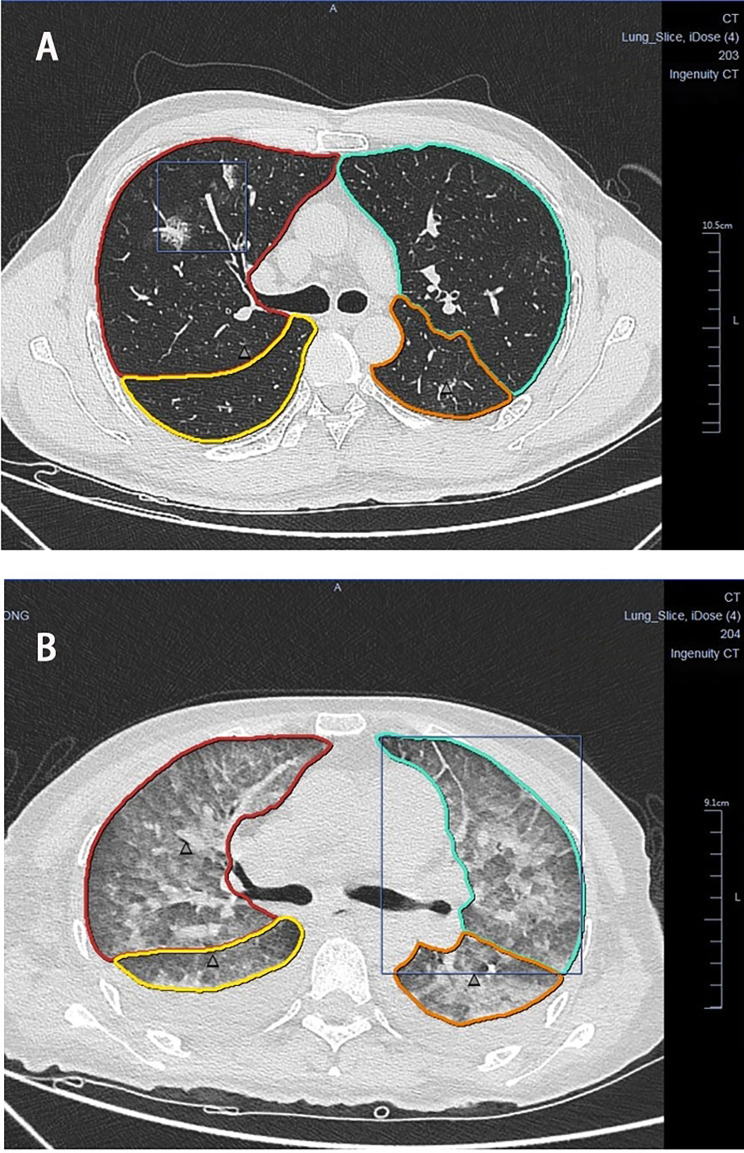
Patients’ chest CTSS: Figure A shows mild pneumonia in patients with scattered multiple ground glass opacities in both lungs, the right upper lobe lesion accounting for 13.9% of lobe volume and a CTSS score of 2. Figure B shows a diffuse exudative consolidation scattered in both lungs, with the left upper lobe lesion accounting for 91.4% of lobe volume and a CTSS score of 5. (**A**) male, 48 years old, a history of diabetes for 3 years, regular medicine with well-controlled blood glucose. (**B**) female, 44 years old, with history of diabetes for 5 years, irregular medication and poor blood glucose control

Since the outbreak of COVID-19, chest CT imaging has played an important role in evaluating infected patients. Pan et al. [22] proposed semiquantitative CTSSs based on the degree of lobar involvement (0–25). We employed the CTSS to evaluate the severity of patients’ images (Fig. 4). Previous research shows that patients with poor blood glucose control have a significantly higher CTSS than those with good blood glucose control [23, 24] because high blood glucose inhibits the immune system and increases the generation of inflammatory factors, causing more severe chest manifestations after the patient contracts viral pneumonia. Statistics show that patients with diabetes have a significantly higher CTSS than those who are not diabetic, and the higher the blood glucose level is on the date of admission, the more severe the lung damage [7, 25, 26]. Our results indicate that blood glucose control is significantly positively correlated to CTSS; patients with poorly controlled blood glucose have higher scores, and lesions commonly involve the lower lobes and peripheral zones of both lungs, further validating that blood glucose affects lung manifestations of the disease. However, blood glucose control and FPG level cannot be treated as independent predictors of clinical severity, and the relationship between blood glucose and COVID-19 requires further exploration. The CTSS can be used to evaluate and predict clinical classification and, in the meantime, offers some accuracy in predicting patient mortality [27], reminding us of the necessity of imaging examination in the diagnosis and treatment of patients with COVID-19. One Iranian study found no significant differences in clinical outcomes and chest CTSSs between patients with diabetes with good and poor blood glucose control [28], which is inconsistent with the findings of this study. This may be attributed to the fact that the former study failed to confirm the diagnosis with test kits and only referred to clinical characteristics and chest imaging manifestation when including patients in the positive group, as well as potential physical differences between Iranian and Chinese patients [29].

Our study also had some limitations. First, the retrospective design with a relatively small sample size may not be adequate to get reliable results. Moreover, COVID-19, as a newly discovered disease, still has many mysteries that remain in dispute, including the assessment of severity. Further large-scale prospective design studies should be conducted to explore the characteristics of COVID-19 and its relationship with diabetes. Moreover, we only included patients with diabetes; the results in patients without diabetes or with other diseases should be explored and the number of patients with COVID-19 infection was not clear in the research hospital.

In summary, chest CT imaging is a reliable testing method for COVID-19 evaluation; the higher the blood glucose level is, the more evident the lung inflammation and the more severe the clinical manifestation. This indicates that clinical patients should first receive a CT scan upon admission to evaluate their initial condition, and imaging appearances should be analysed thoroughly. Targeted treatment should be administered, and publicity and education activities should be implemented aiming to popularise among patients with diabetes the necessity of controlling their blood glucose and encouraging them to make an active effort to keep healthy and follow appropriate diets, thus improving their quality of life.

## Data Availability

All data generated or analyzed during this study are included in this published article.
